# Reviewing the current methods of assessing hydration in athletes

**DOI:** 10.1186/s12970-020-00381-6

**Published:** 2020-10-30

**Authors:** Oliver R. Barley, Dale W. Chapman, Chris R. Abbiss

**Affiliations:** 1 Centre for Exercise and Sports Science Research, School of Medical and Health Sciences, Edith Cowan University, 270 Joondalup Drive, Joondalup, WA 6027 Australia; 2 Performance Support, New South Wales Institute of Sport, Sydney Olympic Park, NSW Australia

**Keywords:** Hydration, Water balance, Fluid, Dehydration, Hypohydration

## Abstract

**Background:**

Despite a substantial body of research, no clear best practice guidelines exist for the assessment of hydration in athletes. Body water is stored in and shifted between different sites throughout the body complicating hydration assessment. This review seeks to highlight the unique strengths and limitations of various hydration assessment methods described in the literature as well as providing best practice guidelines.

**Main body:**

There is a plethora of methods that range in validity and reliability, including complicated and invasive methods (i.e. neutron activation analysis and stable isotope dilution), to moderately invasive blood, urine and salivary variables, progressing to non-invasive metrics such as tear osmolality, body mass, bioimpedance analysis, and sensation of thirst. Any single assessment of hydration status is problematic. Instead, the recommended approach is to use a combination, which have complementary strengths, which increase accuracy and validity. If methods such as salivary variables, urine colour, vital signs and sensation of thirst are utilised in isolation, great care must be taken due to their lack of sensitivity, reliability and/or accuracy. Detailed assessments such as neutron activation and stable isotope dilution analysis are highly accurate but expensive, with significant time delays due to data analysis providing little potential for immediate action. While alternative variables such as hormonal and electrolyte concentration, bioimpedance and tear osmolality require further research to determine their validity and reliability before inclusion into any test battery.

**Conclusion:**

To improve best practice additional comprehensive research is required to further the scientific understanding of evaluating hydration status.

## Background

Homeostatic water balance is essential to life given the role it plays in metabolism, transportation, circulation and temperature regulation [1]. When an individual has a normal body water content they are euhydrated, whereas if they have lower than normal content, they are hypohydrated. The term “dehydration” refers to the process of losing body water but is often used incorrectly to describe hypohydration [2]. Body water is lost through the kidneys, skin (i.e. sweating), respiratory system and gastrointestinal system [3, 4]. Severe hypohydration resulting from excessive sweating during exercise or diarrhoea can have life threatening consequences [1]. Additionally, excessive fluid consumption can pose a risk to health [1]. Hydration status may also influence exercise performance, with a large body of research observing hypohydration to negatively influence exercise performance, in some cases following rehydration [5–7], though this research area has been a topic of debate [8, 9]. Hypohydration impairs exercise through a range of mechanisms, including a reduction in blood plasma/volume, impaired cardiovascular function, muscle blood flow and thermoregulatory capacity [5, 10]. Additionally, hypohydration has been hypothesised to influence neuromuscular function and psychological strain [11]. Given the far-reaching impacts of hydration on function and health, it is unsurprising that there is a large body of research evaluating hydration status in humans [2, 4, 5, 12, 13]. However, despite this body of research no clear best practice guidelines have been agreed upon [2, 14]. In fact, hydration testing is a controversial topic within the scientific community [2, 14]. The controversy stems from several factors including the location and movement of body water, as well as the wide range of available assessment methods. Assessment of hydration in athletes is of particular interest with body water influencing exercise ability, as well as several practices surrounding athletic competition such as reducing body mass in weight-restricted sports [15]. Therefore, the purpose of this review is to provide a practical summary of the potential methods of assessing hydration, their limitations and recommendations for best practice with a focus on athletic populations. Such a review will be useful for practitioners or researchers who are trying to navigate the complicated topic and make practical and well-informed decisions.

### Search strategy

Due to the range of topics explored within this review and the many methods used practically within the field, we elected to take the approach of a narrative review as opposed to a systematic review or meta-analysis which would use a highly technical, specific methodological approach to identify and appraise evidence on hydration assessment [16]. While there are strengths to the systematic approach, a narrative approach allowed for a more flexible structure to provide clarification and nuanced insight into hydration testing with an interpretive and discursive synthesis of the existing literature. Consequently, this narrative appraisal of the literature allows for an interpretive overview, providing reflection and context rather than a formal objective appraisal of hydration testing practices in the constraints of rigidly defined inclusion and exclusion criteria.

For this narrative review, searches were conducted on Google Scholar and Pubmed using the search terms: “hydration”, “dehydration”, “hypohydration”, “body water”, “body fluid”, “assessment”, “testing” and “measurement”. Additionally, the reference lists of found papers were searched to find any additional sources.

### The location of body water

Defining hydration is difficult as the function and storage of fluid throughout the body is complicated. Water makes up around 63% of an adult’s body mass and is regulated in a precise manner [2]. Of this total water volume, approximately 30–35% is intracellular fluid, 20–25% is interstitial fluid, and 5% is retained in plasma [13]. Terms such as “euhydration” “hypohydration” and “dehydration” are typically referring to whole body water content. However, water is stored in many different compartments not only the intracellular, interstitial, and plasma spaces, but also the gastrointestinal tract and bladder [2, 13] and the location of fluid will influence its function (e.g. fluid in the bladder cannot be used for sweating). Fluid located within the interstitial spaces is involved in many vital process at both rest and during exercise [5], likewise cellular fluid volume is critical to cell function and should be a focus when assessing hydration status. It is also important to consider whether the goal is to get a single assessment of hydration status or assess changes over time, as a single measure can be approached differently than assessing changes over time. Changes in total body water (TBW) will not always apply equally to every compartment of fluid throughout the body [2]. This is especially the case for shifts in body position [17], during exercise [5], or during dehydration and rehydration [7]. For example, exercise causes fluid shifts between different compartments, thus complicating subsequent assessments [14]. The range of potential confounders when assessing hydration makes it essential for appropriate standardisation protocols to be followed [18]. Meaningful assessment of hydration status is more difficult than simply assessing changes in TBW. Many assessments involve measuring fluid in specific locations such as urine [19], blood [18], tears [20] or saliva [21], which then provides contrasting information regarding hydration status. Consequently, the location of fluid being assessed is important for informing the methods utilised and interpretations made.

#### Blood variables

Whole blood is essential to many biological processes including the transport of body water and is comprised of erythrocytes, white blood cells, platelets and plasma [5]. Several hydration tests involve the assessment of blood [18] collected using either venepuncture or a simple finger-prick lancet. Venepuncture samples are commonly used to assess blood composition, plasma solutes and hormone concentration [2, 18]. Blood assessments are typically more invasive, expensive and time consuming than several alternative methods of assessing hydration, with an additional minor risk of infection or vein damage [18]. Furthermore, practical limitations can influence the collection and real-time analysis of blood in field-based settings, which should be considered when deciding which methods for assessing hydration are to be used. Regardless, in most situations, blood variables are often more meaningful than their non-invasive counterparts [2].

##### Haematocrit

Dehydration reduces total plasma volume, thus increasing the concentration of blood haematocrit [13, 18]. Haematocrit is typically evaluated from a whole blood sample obtained via finger-prick lancet, capillary tubes and a centrifuge [18, 22]. While haematocrit assessment does not require a phlebotomist, the specific equipment required for analysis does impose a cost barrier [2, 18]. Measures of haematocrit provide an indication of plasma volume and can be used to estimate specific plasma volume loss using theoretical equations if haemoglobin measures are also available [23, 24]. Several factors must be considered when evaluating haematocrit; i) posture, arm position, skin temperature, tourniquet usage and several other factors can all influence reliability [3, 4, 17], ii) haematocrit change from dehydration is less in heat-acclimatised athletes [17], iii) exercise can alter plasma volume for up to 72 h [25], iv) haematocrit levels vary both between- and within-subjects so results must be relative to reliable baseline measurements [3, 5, 22], and, v) haematocrit is not a direct assessment of cellular hydration but instead an assessment of plasma volume [3], the robustness of any inferences to cellular hydration remains unclear.

##### Plasma/serum osmolality

As water is lost from the blood during dehydration the concentration of solutes increases and becomes more hypertonic [26]. This concentration is commonly assessed using freezing point osmometry of serum or plasma [4, 18]. It is important to note that the SI unit for osmolality is “mmol/kg” but many authors use the term “mOsmol/kg” instead, this difference in reported unit of measure should be considered when interpreting the literature. Some laboratories utilize plasma osmolality (P_OSM_) as a gold standard measure of hydration [27], which is a topic of debate [2, 14, 28]. Nevertheless, P_OSM_ is a robust assessment of hydration [5]. There are some important considerations when assessing and interpreting P_OSM_ such as; i) the sensitivity of P_OSM_ to detect mild hypohydration (< 3%) has been debated within the literature [28–31], ii) following exercise, half of all plasma volume lost recovers within one hour even without any fluid ingestion [32]. This phenomenon could result in erroneously concluding a greater magnitude of rehydration occurred than truly did, iii) P_OSM_ is influenced by food as body water shifts from the vasculature into the gut [33], iv) P_OSM_ is highly individual and should be compared to baseline measures as opposed to well-known norms [5, 28], and, v) whilst there is a strong physiological basis for inferring the relationship between P_OSM_ and intracellular hydration [5, 28] it is still not a direct measure of cellular hydration which should be considered when interpreting results.

##### Serum sodium

Fluid shifts during dehydration influence the concentration of electrolytes within the bloodstream [34]. The assessment of electrolytes are used within clinical settings to inform point of care decisions but can also be used for more general or even athlete hydration testing [5, 35]. Sodium in particular can give information on hydration, however there is minimal data exploring the assessment of other electrolytes [5, 18]. Serum sodium is similar to P_OSM_ as it contributes to the majority of P_OSM_ and hence the above limitations of P_OSM_ also apply to serum sodium [5, 36]. While there is evidence to suggest serum sodium is a robust measure there is evidence that the accuracy is less than P_OSM_ [5, 37]. Indeed, the sodium lost in sweat results in serum sodium being less responsive than P_OSM_ [5, 37]. Considering both methods require expensive equipment and trained personnel it seems more practical to use P_OSM_ rather than serum sodium.

##### Hormonal variables

There appears to be a symbiotic relationship between fluid balance and several hormonal factors, such that, changes in hydration results in measurable changes in many hormone levels [22, 38]. Arginine-vasopressin, renin, aldosterone and atriopeptin have been proposed to provide information on hydration status [3, 38, 39]. However, such hormone variables are not typically used in the assessment of hydration. This is likely due to the time and cost associated with analysis and that they are altered by exercise, water immersion and heat acclimation [4, 40–42]. In some cases hormonal variables have been found to be sensitive to changes in hydration levels [43], while other research has found them to be unreliable [5, 44–46]. Recently, a surrogate marker for arginine-vasopressin, copeptin has been used in hydration research with promising results, though further research is required to determine the reliability and validity of the marker across multiple settings [47, 48]. More generally, further research is required to determine the accuracy and validity of hormonal responses to hydration status prior to their inclusion as a commonplace assessment of hydration status.

##### Summary of blood variables

Blood is widely considered as a reliable fluid to assess hydration [2, 5]. Of the available variables, plasma/serum osmolality is the most reliable and valid [5]. Many variables typically associated with blood may be assessable in other bodily fluids. For example, electrolyte or hormone concentrations could theoretically be assessed in other fluids such as urine or tears, but further research is required to investigate the accuracy and sensitivity of such methods. Blood collection is invasive which may serve as a barrier to utilisation in practical settings, hence other fluids such as urine, saliva or tears are worthy of consideration. However, when assessing athletes in laboratory conditions blood-based assessments of hydration should be utilised due to their validity and reliability as described. In athletic settings it is also important to consider that blood variables will take time to process depending on the availability and proximity of the required equipment, which may lead to delays during important point of care decisions.

#### Urine variables

Urine is comprised of water and several other substances which increase in concentration as the volume of water decreases [49]. Urine output is influenced by fluid intake, diet (e.g. electrolyte intake), drugs (e.g. alcohol or caffeine) and/or illness (e.g. diabetes or kidney disease) [49]. As a result, previous ingestion or medical conditions must be accounted for when assessing hydration via urine. Urinary hydration assessment methods include urine specific gravity (USG), urine osmolality (U_OSM_), urine colour (U_COL_) and urine volume [19]. USG is assessed by placing a small volume of urine onto a refractometer and the urine density is compared to double distilled water (density = 1.000). A result greater than 1.020 is typically considered hypohydrated [13, 19]. U_OSM_ assesses the total solute content of the urine and involves taking ~ 20 μL of urine and assessing its freezing point depression [19]. It is possible to assess urine osmolality without an osmometer and instead using a hand-held conductivity meter [50], however this method is actually and extrapolation from USG. Using this alternative method, a urine osmolality over 700 mmol/kg is typically considered dehydrated [51]. Urine colour is a subjective evaluation of urochrome in the urine and uses a Likert scale. When more water is excreted, urine colour becomes paler and conversely becomes darker as less water is excreted [19]. Urine assessments are less invasive than blood variables, and with the exception of U_OSM_ they are relatively inexpensive [2, 19].

There are several considerations when implementing urinary hydration assessments; i) a urine sample reflects all urine in the bladder since the previous void, thus the timing since the last void will influence results [26], ii) ingesting hypotonic fluids results in water being excreted before the intracellular and extracellular fluids equilibrate, this can result in erroneous urine results indicating euhydration [2, 52], iii) when assessing acute dehydration and rehydration, urine variables poorly correlate to more robust measures such as P_OSM_ due to hormonal changes during rehydration influencing the reabsorption of water and electrolytes [11, 26, 53], iv) previous research investigating the accuracy of urinary assessments is mixed, with research reporting it to be robust [30, 52, 54] while other research indicates the opposite [15, 55–58], v) as with P_OSM,_ there is a biological variation between individuals and hence, use of single cut-off points as opposed to comparisons with baseline measures is likely to produce erroneous results [5, 28], vi) previous research has suggested the use of single spot measures be excluded entirely due to the large degree of potential confounding factors and questionable normative values [59], vii) the urinary excretion rate has identified as a potential confounder of concentration-based assessments which should be accounted for where possible, and, viii) urinary measures represent the renal response to fluid homeostasis and not real-time hydration status at a cellular level [60].

Based on this information, urinary measures should be used with caution and in conjunction with other methods. The convenience of urinary assessments makes them appealing in both laboratory and field-based settings. However, single cut-off limits should be avoided where possible and instead comparisons to within-individual changes (i.e. baseline measures) implemented [59]. Of the urine variables, USG and U_OSM_ appear the most reliable [5, 54]. However, given the limitations of urine as a fluid in general, caution should be applied if researchers intend to utilise these as a substitute for blood assessments. Additionally, urinary measures should be avoided in cases of rapid rehydration such as those occurring post weigh-ins during weight-restricted sports [7, 15, 54]. Due to the ease, speed of collection and analysis for urinary variables they are appealing for use in athletic settings but must be used carefully to avoid erroneous conclusions. For example, combining urinary measures with gross measures of body mass and a blood marker will vastly improve their practical usage.

#### Saliva variables

Saliva osmolality and flow rate can be noninvasively sampled to estimate hydration status. Both salivary flow rate and osmolality respond to exercise-heat stress and fluid restriction but the variation between individuals is large [5, 61–63]. In fact, the day-to-day variability of saliva osmolality has been reported to be almost 10 times greater than P_OSM_, body mass, or even USG [21, 64]. Salivary osmolality is influenced by fluid and food ingestion with previous research reporting a 10 s mouth rinse with water to influence results for up to 15 min [21]. Exercise also influences salivary sodium, potassium and protein which could confound measures of salivary osmolality [65, 66]. Considering the poor reliability and large number of confounding factors associated with salivary variables, the use of this technique is questionable. Furthermore, use of salivary variables in athletic settings appears inappropriate due to the high likelihood that athletes will be exercising and/or ingesting fluids. Other potential variables such as salivary electrolytes or hormones may provide a more reliable assessment of hydration, however, systematic examination is required to confirm or refute the validity, sensitivity and reliability in this setting.

#### Tear osmolality

A recent method of estimating hydration status involves assessing fluid of the eye. Tear osmolality has emerged as a strong candidate for hydration assessment [67, 68]. Indeed, tear osmolality closely correlates with P_OSM_ with the relationship being stronger than USG [20]. However, literature has reported a large variability of tear osmolality potentially due to evaporation and differences in collection techniques [20, 67]. Recently, a non-invasive tear collection and analysing device has provided a potential solution for the disparate collection techniques [67]. However, a recent study using the non-invasive tear collection and analysing device found that while tear osmolality did change following exercise-induced fluid loss, it did not correlate to other laboratory hydration measures including plasma osmolality and urine specific gravity [69]. Further research using the non-invasive tear collection and analysing device reported an inability to reliably detect mild dehydration [70]. Tear osmolality may provide a non-invasive alternative to assess hydration, but further research is required to understand its reliability, precision, limitations and ideal collection techniques. If tear osmolality can be demonstrated to be appropriate and robust, the technique will be appealing in athletic settings due to possibly lower participant burden and high scientific accuracy.

#### Stable isotope dilution

Stable isotope dilution involves measuring trace amounts of a particular isotope (usually deuterium oxide, ^2^H_2_O) in blood or urine and calculating the TBW [71]. The calculation of TBW is based on the dilution principle, with previous research reporting such methods as highly accurate [72]. After baseline measures are collected the subject ingests an oral solution containing a known amount of the chosen isotope. Multiple samples are then collected over the following hours to determine TBW [73, 74]. Such methods require costly equipment, significant periods of time and technical expertise [2]. While there is a strong body of research supporting the accuracy of stable isotope dilution [4, 72] it is not a direct measure of cellular hydration but rather, is based on the assumption that the isotope will distribute equally throughout extracellular and intracellular fluids, which is unverified [2]. Additionally, it takes longer for isotopes to equilibrate within urine than blood so more time for data collection will be required for urine [72]. Stable isotope dilution may a highly accurate method of assessing hydration status, but due to technical limitations of the technique it is not realistic to use in many applied settings, though it may have uses in controlled laboratory settings. Additionally, due to the time taken to complete analysis it is not a practical real time assessment of change in hydration status. However, due to its high accuracy it is an appropriate measure of hydration status under highly-controlled conditions.

### Gross assessments of hydration status

While hydration can be assessed using several fluids throughout the body, there are also several assessments that take more gross estimates of hydration status such as body mass, vital signs and sensations of thirst, bioimpedance, dual-energy X-ray absorptiometry and neutron activation analysis [2]. These methods potentially alleviate the issue of using fluid from one compartment to predict the hydration status of the entire body. However, gross assessments of hydration status will likely be unable to determine fluid shifts within the body, which has practical implications.

#### Body mass

Changes in body mass can be used to estimate the volume of water lost during exercise and/or thermal exposure, so long as fluid and food intake and excretion via urine and faeces are considered [22]. Assuming any change in body mass is entirely due to changes in body water then 1 g of body mass should equate to 1 ml of water [22]. Such assessment can provide a general indication of change in whole body fluid content. A range of equipment can assess body mass including underwater weighing, air displacement or floor scales [70, 75]. Equipment accuracy varies, with floor scales having varying reliability and accuracy dependant on the model of scale and method of measurement used [76, 77]. The process of estimating whole body sweat loss via body mass is far more complicated than one may expect. Indeed, body mass assessments may be confounded by time of day, food/fluid consumption, sweat composition, respiratory water loss, exercise-induced substrate utilisation and metabolic water production [22, 60, 77, 78]. The longer the period of time between measures, the greater the difficulty in maintaining appropriate controls to ensure body mass changes relate predominantly to changes in hydration. In an effort to account for the potential confounders, Cheuvront and Kenefick [77] have presented an equation for accurately determining change in body mass:

$$ \Delta  \mathrm{BM}=\left({\mathrm{H}}_2{\mathrm{O}}_{\mathrm{drink}}+{\mathrm{H}}_2{\mathrm{O}}_{\mathrm{food}}\right)-\left({\mathrm{H}}_2{\mathrm{O}}_{\mathrm{urine}}+{\mathrm{H}}_2{\mathrm{O}}_{\mathrm{feces}}+{\mathrm{H}}_2{\mathrm{O}}_{\mathrm{skin}}+{\mathrm{H}}_2{\mathrm{O}}_{\mathrm{resp}}\right)+\left({\mathrm{solids}}_{\mathrm{in}}-{\mathrm{solids}}_{\mathrm{out}}\right)+\left({\mathrm{gases}}_{\mathrm{in}}-{\mathrm{gases}}_{\mathrm{out}}\right) $$

When adequate controls (i.e. time of day, food intake and bowel content) are maintained, changes in body mass can provide an indication of whole body hydration for up to 2 weeks, assuming relatively consistent energy balance and that the subject is not growing as a result of youth (i.e. puberty) [4, 79, 80]. Over long periods of time, changes to body composition reduce the accuracy of body mass hydration assessments [79]. Under the correct conditions and in conjunction with other assessments of hydration, body mass provides useful information on hydration status, especially within shorter periods of time (i.e. ≤ 24 h).

#### Vital signs and sensation of thirst

Hypohydration affects the cardiovascular system which can be used to assess hydration status. Plasma volume reduction influences total blood volume and theoretically blood pressure [81]. However, blood pressure is a poor diagnostic tool for hydration assessment due to how robustly it is regulated [5, 15, 70, 82]. The reduction of blood volume resulting from hypohydration also reduces stroke volume and results in increased resting and submaximal heart rates [81, 83]. As a result, change in heart rate from sitting to standing can be used to evaluate the degree of hypohydration [5, 82]. Unfortunately, change in heart rate from sitting to standing has shown poor sensitivity and weak overall accuracy [84], though there is evidence to suggest it may be able to detect extracellular dehydration [70]. Heart rate is influenced by a wide range of factors outside of hydration status, thus making it problematic to assess changes in hydration status [5, 84]. Physical signs such as sunken eyes, capillary refill time and skin turgor have also been shown to be highly inaccurate in diagnostic settings [4, 5]. Thirst sensation may also be used to assess hydration status using various assessment scales [85]. Subjective sensation of thirst is typically assessed using a Likert scale ranging from 1 (not at all thirsty) to 9 (very, very thirsty) [86], while a rating of between 3 and 5 is typically used to identify mild hypohydration [85]. Recent research has reported that sensation of thirst can accurately detect mild dehydration [70]. However, perception of thirst is influenced by palatability, time allowed for fluid consumption, gastric distention, age, gender and heat acclimation status [87–89]. It is also possible that in athletic settings where athletes may wish to hide potential hypohydration (e.g. during rapid weight loss in combat sports) they could intentionally provide inaccurate results. Thirst sensation lacks the precision for detailed evaluation of hydration status but could provide a useful approximation [2, 70]. While vital signs and sensation of thirst may be important in understanding the physiological or perceptual responses associated with hydration, they themselves provide limited information on hydration status but may be of use in conjunction with other more robust assessments.

#### Dual-energy X-ray absorptiometry (DXA)

Dual-energy X-Ray absorptiometry (DXA) is commonly used as a measure of body composition with a focus on bone mineral density [90]. Though not the primary use of a DXA, it can be used to gather information on TBW. Indeed, body water is located primarily within the lean body mass component of the DXA output [90, 91]. At rest and within short time-periods, changes in lean body mass measured by a DXA will be the result of changes in TBW [90]. However, exercise and food ingestion alter factors within the lean body mass measurement, namely the concentration of muscle glycogen [91] which may confound measures in athletic populations. Additionally, the use of multiple measures to detect change would require the exposure of athletes to multiple bouts of radiation, albeit a small amount [92].

#### Bioimpedance

Bioelectrical impedance analysis can quickly and non-invasively assess TBW. It involves a low alternating current being directed through the body and the resistance of the current measured to estimate TBW [93]. Measurement precision can be affected by subject posture, skin temperature, electrolyte balance, ingestion of food, intense physical activity, alcohol ingestion and protein malnutrition [3, 94]. Typical error for TBW assessment ranges from 1.5–2.5 kg for bioelectrical impedance analysis whereas more advanced bioelectrical impedance spectroscopy, is more accurate and can predict extracellular and intracellular water [18]. However, predictions of extracellular and intracellular water are highly theoretical and further research is needed determine the accuracy of such calculations [2, 22]. The accuracy of bioimpedance techniques is unclear with research indicating changes in body fluid volume and tonicity can influence accuracy [18, 22]. As a result of the potential confounders and lack of scientific verification of using bioimpedance techniques to assess hydration status, previous research has discouraged its use when monitoring acute changes in hydration status [3, 4]. However, bioimpedance techniques have potential but further research is required to determine the precision and reliability of bioimpedance before advocacy for inclusion in a hydration testing battery. If bioimpedance can be demonstrated to be robust it will be appealing in athletic settings due to the limited burden and convenience of the data collection/analysis.

#### Neutron activation analysis

Neutron activation analysis uses radiation detectors to measure total body chloride, potassium and sodium following exposure to a neutron field and using the results to determine extracellular and intracellular volume [2, 71]. The scan typically takes one hour to complete and has been reported to be a highly accurate measure of TBW [2, 71, 95]. Neutron activation analysis only estimates TBW based on electrolytes throughout the body, as opposed to directly measuring it [2]. Neutron activation analysis requires costly equipment [71], significant periods of time and technical expertise to complete, with the additional issue of radiation exposure as part of the assessment [2, 71]. Due to the resources required to conduct neutron activation analysis and the potential risk to participant’s wellbeing it will not be appropriate in most practical settings. However, like stable isotope dilution, neutron activation analysis is highly accurate and could be important in assessing the validity and accuracy of other methods.

#### Developing recommendations

The purpose of this review was to review the methods of assessing human hydration and provide recommendations in athletic settings. The assessment of human hydration is a complicated topic and there is no single flawless method of assessing hydration status (Table 1). The accuracy and validity of differing measures of hydration will vary depending on the situation. The first thing to consider is the objective of the assessment protocol. Assessments of hydration status can investigate specific locations or the body as a whole. There may be scenarios where the assessment of a singular location is the objective, but in most cases, investigators will be aiming to get an indication of whole body hydration status, which the following section will focus on.

**Table 1:** Characteristics of methods for assessing hydration

Hydration assessment/variable	Hydrated upper limit	Limitations^a^	Precision/reliability	Cost	Invasiveness	Administrator skill required	Time required
Stable isotope dilution	N/A	Expensive and time consuming	5	5	5	5	5
Neutron activation analysis	N/A	Expensive and time consuming	5	5	5	5	5
Haematocrit	< 2% change	Influenced by many confounding factors	3	3	3	3	2
Plasma/serum osmolality	< 296 mmol/kg	Expensive and invasive	4	4	4	4	2
Serum sodium concentration	< 145 mEq/L	Expensive and invasive	3	4	4	4	2
Hormonal variables	N/A	Expensive and undetermined validity/reliability	2	4	4	5	4
Urine specific gravity	< 1.020 SG	Many confounding factors	2	2	2	2	1
Urine osmolality	< 700 mmol/kg	Many confounding factors	2	3	2	3	2
Urine colour	< 4	Many confounding factors	1	1	2	1	1
Saliva osmolality	< 61 mmol/kg	Questionable reliability	1	3	2	3	2
Tear osmolality	< 310 mmol/kg	Undetermined validity/reliability	3	3	2	3	2
Body mass	< 2% change	Many confounding factors and only works within limited time periods	4	1	1	1	1
Bioimpedance	N/A	Many confounding factors and questionable reliability	3	2	1	1	1
Vital signs^a^	N/A	Questionably validity/reliability	1	1	1	2	1
Sensation of thirst	< 3 (scale of 1–9)	Low sensitivity	1	1	1	1	1

Rating: 1 = lowest, 5 = highest. N/A = Clear cut-off has not been defined or do not apply. ^a^See the respective section for more details

**Fig. 1:**
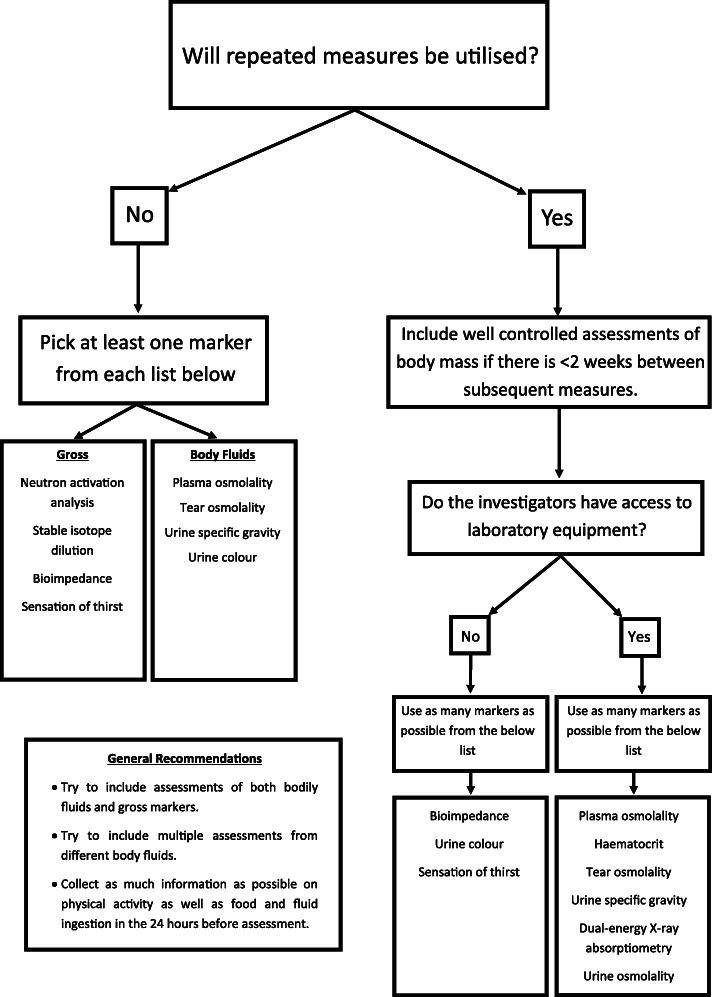
A flowchart for designing a comprehensive hydration testing protocol

When designing a whole body hydration testing protocol, many things must be considered including desired accuracy/validity, cost, location or area of interest, practicality and sample collection restraints (all explored in the sections above) (Table 2). Investigators also need to consider whether repeated measures will be utilised (Fig. 1). When repeated measures will not be utilised, it is important to apply appropriate gross assessments (i.e. neutron activation analysis, stable isotope dilution, bioimpedance and sensation of thirst) and those derived from bodily fluids (i.e. plasma osmolality lower than 296 mmol/kg, tear osmolality under 310 mmol/kg, U_OSM_ below 700 mmol/kg and USG under 1.020 [20, 27, 28, 51]) using appropriate cut-offs with caution, especially in the case of urinary markers [59] (Fig. 1; Table 2). Though the use of P_OSM_ is clearly the most appropriate when only a single measure is being utilised [28]. When repeated measures are utilised, well-controlled assessments of body mass should be employed wherever possible. When subjects are engaging in exercise the potential influence of substrate utilisation and metabolic water production on body mass must be considered. In cases where the investigators have access to laboratory equipment, they should try to implement as many assessments as possible from the following list: plasma osmolality, haematocrit, tear osmolality, USG, DXA, U_OSM_ (Fig. 1). Without access to laboratory equipment, investigators should try to use bioimpedance, urine colour and sensation of thirst while considering that the validity of the battery will be lower than a laboratory-based protocol (Fig. 1). Where possible, assessments of different bodily fluids will be valuable. While these guidelines are based on the current body of literature, it is important to update best practices as research progresses.

**Table 2:** Example Hydration Testing Protocols for Athletes

	Individual A (Competitive boxer who has just made weight)	Individual B (Cricket player playing in a 5-day test match)	Individual C (Marathon runner preparing for a major competition with two prior qualifying events)
Logistics	• The weigh-ins are conducted at a stadium by the regulating commission away from laboratory equipment 24 h before the event. • There was no availability for pre-weight loss measures • Athlete needs to begin rehydrating as soon as possible • Coach and athlete preference is to avoid blood draws so close to competition	• The competition situation is located away from a laboratory • Measures can be taken before and after the day’s play as well as between sessions of play • The player will be ingesting fluids throughout the day • Antecubital blood draws are not permitted	• Historical environmental evidence suggests that the athlete will be competing in hot and humid environments, as such the decision taken by the coach and support team is to implement a heat acclimation strategy • Events are separated by approximately 14 weeks, beginning 7 weeks before the event, a 6 week heat acclimation protocol inclusive of adaptation monitoring will be instigated for a 7 day taper into the event • In addition, support staff wish to know if the competition hydration strategy implemented at each event is suitable • At each event, testing will take place 2 h prior, as soon as practically possible after finishing and 1 h this post race collection
Suggested tests	• Urine specific gravity • Body mass recovery between the weigh-in and the competition • Tear osmolality	• Urine specific gravity • Sensation of thirst • Body mass change	• Urine specific gravity • Serum osmolality • Haematocrit • Tear osmolality • Sensation of thirst • Body mass
Reasoning	The above protocol involves testing two different body fluids which can be easily assessed at an external location. Additionally, there is a gross marker of hydration (body mass change), which can help provide a more complete hydration picture. Importantly if too much body mass (> 15%) was regained by the competition, then the bout could be cancelled, or the athlete could be required to move up a weight class for their next competition. Sensation of thirst was not selected as the athlete may not answer honestly in an effort to mask dehydration.	The above protocol requires only involves a single (convenient) measure of a bodily fluid but includes two gross markers of hydration status. When beginning monitoring prior to the first session of play, the protocol relies on USG and sensation of thirst, whereas changes in body mass can be used throughout the day to prescribe fluid intake. The waking body mass from the first day can then also be used to interpret subsequent morning measurements, and between sessions of play. Provided, the fluid intake during the session is measured, this does allow for a highly accurate understanding of hydration status change.	The above protocol involves the assessment of three different bodily fluids and two gross markers of hydration status. This combination will provide a more complete assessment of whole body hydration and consistency of assessment technique from the acclimation period through to event. The pre-event testing protocol can be completed within 30 min enabling recommendations to be quickly given and responses implemented prior to competition if necessary. The post-event testing will be able to confirm if the hydration strategies used during the event were sufficient and ideally not a limiting factor to performance.
Considerations	While the suggested protocol involves the assessment of multiple bodily fluids, the reliability of spot urine assessments is questionable and the validity/reliability behind tear osmolality is still unclear. However, if the fluid markers indicate hypohydration and a large amount of body mass is regained between weigh-in and competition, then the athlete’s support staff can be confident that they were significantly hypohydrated at the weigh-in and make the decision whether to cancel the bout or strongly recommend that the athlete change weight classes for future bouts.	Application of this method is best in a well-controlled situation, with the most important factor being controlling/measuring all food/fluid that athletes take in across the multiple days of competition. In situations where some measures suggest hypohydration and others do not, it is best to encourage the athlete to ingest fluids.	By implementing acclimation strategies prior to each event, training load, individual physiological adaptation, and familiarity with testing processes can continue to be refined. Continual education of the athlete can be achieved during each acclimation period and qualification event to the signs and extent of any indication of hypohydration, leading to a more self-aware athlete for the need to ingest additional fluids. Support staff should seek to take note of the relationship between the less invasive markers and those derived from invasive measures to better contextualise individual response. Understanding this individual response for each marker and their relationship can allow for the possibility at the major event, to remove the invasive blood markers and rely on the non-invasive tests in an effort to reduce any potential influence and anxiety the athlete may hold for their performance.

Following standardised protocols alongside carefully selecting tests in context of the specific scenario is essential. Regardless of the method utilised results should be compared to changes within an individual to control for biological variation between humans due to known differences in fluid retention volumes and sites [5, 64]. The most accurate measures of assessing hydration status (i.e. stable isotope dilution and neutron activation analysis) require equipment that the majority of investigators will not have access to, and even when they do, due to the time required to complete the analysis it is not realistic to use them to assess change in hydration. However, if the time and resources are available, they are likely to provide highly accurate measures. Blood variables (especially P_OSM_) are typically more accurate than other assessments of body fluids and while they do require specialised equipment and skills, most laboratories will have access to the appropriate resources. Blood variables such as P_OSM_, serum sodium and haematocrit can be conducted relatively quickly, allowing them to conveniently assess changes in hydration which may inform point of care decisions. Without access to advanced laboratory equipment, urine specific gravity is next best available option but must be used with careful consideration of its limitations. Other variables such as bioimpedance and tear osmolality should be used with caution until further comprehensive research is conducted to better understand their reliability and validity as assessments of hydration status (Table 1). However, in athletic settings the primary limitations will be logistical in nature such as limited access to equipment, expertise or time (Table 2). In cases when logistical issues arise, it is important to try to work within such limitations to select the best testing battery possible and then interpret the results through a critical lens (Fig. 1; Table 2).

We strongly recommend the use of multiple measures of hydration status simultaneously for three reasons: i) no single measure of hydration is without limitations, nor is a comprehensive measure of intra- and extra-cellular hydration, so multiple assessment methods increases accuracy and validity, ii) multiple assessments reduce the likelihood of incorrect categorisation of hydration (i.e. hypo, hyper or euhydrated) due to measurement error, and, iii) different methods of hydration assessment evaluate fluid in different parts of the body which all interact with each other (intracellular, extracellular and both in the same variable) so it is important to use multiple methods (both gross and body fluids) to give the investigator a more comprehensive picture of where fluid is retained within the body. However, even in cases where multiple assessments are used with careful consideration of their limitations it is important to acknowledge there is currently no direct assessment of intra- and extra-cellular hydration and the current assessments are estimates only of the location of fluid within the body. Finally, physiological changes associated with variations in hydration are not completely understood and the effects of hydration on both performance and health are more complicated than simply the location and total volume of body water.

### Future research directions

Future research should aim to better understand the movement of fluid between different compartments in the body and how to best assess the hydration of such compartments. Development of an assessment of hydration that can directly assesses intra- and extra-cellular hydration would provide valuable results when trying to understand how hypohydration influences bodily function instead of simply looking at total body water. Previous research has explored the concept of assessing hydration via a biopsy of muscle tissue which could provide a more direct measure of skeletal muscle hydration, however more research is needed to better understand the accuracy and reliability of any such method [96–98]. A muscle biopsy would be highly invasive and uncomfortable for subjects so its best usage may be as a reference assessment to test the accuracy of less invasive measures. Development of more accurate but less invasive measures of hydration status is important for cases where blood assessments are impractical. More research investigating the accuracy, reliability, and validity of tear osmolality and bioimpedance is warranted. Ultrasound technology may also have the potential to provide information about hydration status but the technique is in its infancy and more research is required [5, 99]. Finally, many assessments have not had their accuracy and reliability assessed in the case of rapid dehydration or rehydration which may significantly influence potential results, especially in cases where liquids lacking electrolytes are ingested for recovery [15].

## Conclusions

While previous research has examined the assessment of hydration status in athletes, this review provides a novel set of guidelines for developing an assessment battery of hydration status for different situations. There are a wide range of methods to assess hydration status. Some methods are supported by a large body of scientific research while others have little supporting evidence. Researchers should aim to systematically fill the gaps in research while pursuing new avenues of hydration assessment. Practitioners and researchers who are aiming to assess hydration status need careful consideration when selecting a hydration status testing protocol to get valid and meaningful data. Additionally, no assessment of hydration status is without limitations so investigators should be cautious in the collection and interpretation of data. Better understanding hydration assessment will have important applications in both clinical and athletic settings.

## Data Availability

Not applicable.

## Abbreviations

TBW: Total body water
P_OSM_: Plasma osmolality
USG: Urine specific gravity
U_OSM_: Urine osmolality
U_COL_: Urine colour
DXA: Dual-energy X-ray absorptiometry
